# Comparison of the Bulut Index-Beta method and Global Health Security Index: results from the world’s countries

**DOI:** 10.55730/1300-0144.5854

**Published:** 2024-06-01

**Authors:** Tevfik BULUT, Mehmet TOP, Murat ATAN, Burkay GENÇ

**Affiliations:** 1 Department of Health Management, Faculty of Economics and Administrative Sciences, Hacettepe University, Ankara, Turkiye; 2 Department of Econometrics, Faculty of Economics and Administrative Sciences, Ankara Hacı Bayram Veli University, Ankara, Turkiye; 3 Department of Computer Engineering, Faculty of Engineering, Hacettepe University, Ankara, Turkiye

**Keywords:** Bulut Index-Beta, BI-β, COVID-19, Global Health Security Index, GHSI, pandemic

## Abstract

**Background/aim:**

The Global Health Security Index (GHSI), which is used to assess the global health security preparedness levels of countries, and the Bulut Index-Beta (BI-β) method, developed as a multicriteria decision-making method, were compared in terms of global health security in the context of the world’s countries. It was aimed to demonstrate the feasibility of the BI-β method by testing it on GHSI datasets and contribute to the methodological development of the GHSI.

**Materials and methods:**

The datasets used in this study were the publicly available GHSI datasets, which allow for comparative evaluations of countries. The BI-β method and GHSI were used to compare countries in terms of global health security.

**Results:**

In 2021, the top three best-performing countries in terms of global health security were the United States (BI-β = 85.46), Australia (BI-β = 82.53), and the United Kingdom (BI-β = 82.29). For 2019, the United States (BI-β = 85.44) and Australia (BI-β = 81.69) had the same ranks as in 2021, but the United Kingdom (BI-β = 76.63) dropped to 9th place. There was a statistically significant positive weak monotonic relationship between BI-β and GHSI rankings.

**Conclusion:**

Since the GHSI scoring system is not consistent or questionable, the weighting process needs to be objectively reconsidered and the reasons for the weighting process need to be explained. The GHSI was conceptualized based on a narrow and technical framework. Therefore, it is recommended that the social and political determinants of public health be taken into account in the GHSI. On the other hand, the BI-β method can be easily used in solving other multicriteria decision-making problems, especially in public health areas such as global health security.

## 1. Introduction

In this study, the Global Health Security Index (GHSI), covering the years 2019 and 2021, and the Bulut Index-Beta (BI-β), developed as a multicriteria decision-making method (MCDM), are compared in the context of the world’s countries. Thus, it is aimed to demonstrate the feasibility of the BI-β method by testing it on GHSI data assessing the global health security preparedness levels of countries and contribute to the methodological development of the GHSI. The secondary objective of this study is to propose an application algorithm for increasing the accessibility of the BI-β method in the R programming language. It is anticipated that the findings to be obtained using the BI-β method will contribute to the development of the GHSI by providing a different perspective on the methodological framework of the GHSI. The BI-β method can be applied in solving multicriteria decision-making problems regardless of the sector using the application algorithm, written on small-scale and large-scale datasets. In the health sector, where big data and large datasets are generated, the BI-β method has potential areas of use in the evaluation of the financial performance of hospitals, quality evaluations of units within hospitals, hospital location selection problems, the purchasing of materials and equipment for hospitals, evaluations of marketing strategies in the health sector, and evaluations of primary and secondary healthcare institutions. When the relevant literature is reviewed, there are very few methodological studies on comparative evaluations of the GHSI with MCDM approaches. At the same time, there has been no methodological study to date comparing the BI-β with the GHSI. These points constitute the motivation of this study.

Global health, which has become an important issue on the international policy agenda due to global health crises such as the COVID-19 pandemic, reveals that public health has gained an international dimension beyond national borders [1]. Today, with increasing mobility and globalization, public health security has become more important than ever and relevant measures have been taken with the participation of international institutions and organizations [2]. In this context, the foundation of an effective global outbreak warning and response system was built on the concept of cooperation by the World Health Organization (WHO) in 1996 with the participation of many international institutions and organizations. The global management of outbreaks was facilitated by establishing systematic mechanisms for gathering outbreak intelligence and verifying the existence of outbreaks.^1^ It was accepted that no single organization or institution alone has the capacity to respond effectively to outbreaks with the Global Outbreak Alert and Response Framework (GOARN) document published by the WHO after a meeting held in Geneva in April 2000. In the same document, it was emphasized that the fight against epidemics requires the effective technical partnership of national and international institutions and networks, and that political, economic, and environmental conditions in epidemics should be addressed in a holistic and sustainable manner [1,3].

Public health security is defined as proactive and reactive actions to strengthen countries’ defenses against acute public health events that endanger public health. In global public health security, public health security is extended to broader geographical regions and international communities. Global public health security covers a wide range of issues, including the health consequences of human behavior, meteorological events, infectious diseases, natural disasters, and man-made disasters.^1^

Composite indices are among the tools frequently used by decision-makers to present a holistic picture by simplifying issues that are complex and sometimes difficult to understand. Composite indices, which have proven to be useful in comparing country performances, summarize complex and multidimensional issues and provide support to decision-makers [4]. The Global Health Security Index (GHSI), which is structured as a composite index, aims to reveal and improve countries’ levels of preparedness and abilities to respond to infectious disease threats that may become epidemics or pandemics around the world. The GHSI provides a transparent and comprehensive assessment of global health security in 195 countries. As a project of the Nuclear Threat Initiative (NTI) and the Johns Hopkins Center for Health Security, the GHSI was developed together with Economist Impact. It aims to develop and promote measurable indicators of national health security and improve the international capability to combat infectious disease threats that could lead to international outbreaks and pandemics [5].

When studies conducted in the field of public health are examined, there are many studies revealing the value of the GHSI in the literature and addressing the question of whether it is effective in predicting epidemics [6–15]. Among those studies, some studies found weak associations between GHSI scores and certain COVID-19 pandemic findings, while other studies found the expected associations between GHSI scores and COVID-19 outcomes. One study examined the practical value of the GHSI, uses of the GHSI in helping policymakers and practitioners maximize its benefits, and the methodology underpinning country scores and rankings in the GHSI [6]. Another study analyzed the relationship between the performance of 36 Organisation for Economic Co-operation and Development (OECD) member countries during the pandemic and their GHSI scores. In that study, it was revealed that there was a discrepancy between GHSI scores and the actual performance of countries during the pandemic [7]. In a study evaluating the external validity of the 2019 GHSI, the relationships between global infectious disease outcomes and GHSI scores were determined by linear regression models. It was stated that although the GHSI is a valid measurement tool for assessing health security, the index has potential deficiencies in terms of global health security capabilities and is insufficient in identifying anthropogenic threats. For these reasons, it was emphasized in that study that the GHSI should be more widely accepted [8]. Another study criticized the scoring system, the weighting of indicators, and the validity of the GHSI. The criticisms of that study pertained to the haphazard weighting of indicators in the GHSI and the fact that the GHSI does not take into account the realities of some low- and middle-income countries [9]. In a study in which the effectiveness of COVID-19 vaccine rollout by OECD countries was estimated with the GHSI, it was argued that the preparedness of OECD countries for infectious disease threats was not accurately estimated by the GHSI [10]. Another study emphasized that there is little correlation between GHSI results and countries’ actual COVID-19 experiences [11]. In another study, the COVID-19 results of 195 countries were analyzed in the context of the 2019 GHSI. It was emphasized that the COVID-19 outbreak revealed many of the capacity deficiencies stated in the 2019 GHSI and that no country was adequately prepared for such a pandemic. However, the GHSI provided an opportunity for countries to identify and address their capacity deficiencies [12]. In a study conducted to demonstrate the effectiveness of the GHSI, the relationship between the GHSI and the COVID-19 outbreak was analyzed. That study concluded that the GHSI had limited value in assessing the capacity of countries to respond to the COVID-19 pandemic [13]. In a cross-sectional study, the relationships between the dependent variables of COVID-19 cases and deaths for 92 countries and independent variables including the GHSI were revealed. According to the findings, the GHSI was positively correlated with COVID-19 cases and deaths [14]. In another study, possible relationships between COVID-19 cases, deaths, and vaccination and countries’ GHSI scores were examined. According to that study, higher GHSI scores of countries do not mean that those countries responded optimally to the COVID-19 outbreak. The study also emphasized that the world is not sufficiently prepared for future major pandemics and that the methodology of the GHSI should be revised to take into account deficiencies in health systems.

On the other hand, there are also studies in the literature [16–18], albeit quite limited in number, in which the GHSI is used together with MCDM approaches to evaluate countries and hybrid approaches are proposed using MCDMs. In one such study, the performances of the health systems of 195 countries were evaluated using six indicators from the health dimension of the 2019 GHSI by means of MCDMs. In that study, the indicators from the health category of the 2019 GHSI were first determined as decision criteria and then countries from the GHSI were determined as decision alternatives in the evaluation of health systems. One of the motivations of that study was the problems encountered in determining the criteria weights in the GHSI and the question of how to generate more added value from the GHSI. A hybrid model for the evaluation and improvement of health systems was proposed using the CRITIC method for weighting the decision criteria and the modified VIKOR method for evaluating decision alternatives according to the decision criteria. The results obtained from the modified VIKOR method were then compared with the traditional VIKOR, TOPSIS, grey relational analysis (GRA), and COPRAS methods. The relationship between the rankings obtained from the modified VIKOR, TOPSIS, GRA and COPRAS methods was measured with the Spearman rank correlation coefficient. According to the findings, there was no statistically significant relationship between the modified VIKOR method and other methods. This result was attributed to the fact that the theoretical concept of the modified VIKOR method is different from those of the other methods [16]. In another study, the health security performances of EU member states were measured with multiattribute ideal–real comparative analysis (MAIRCA), which is one of the MCDM approaches, over datasets belonging to the dimensions of the 2019 GHSI. In the same study, the relationship between the health safety performance values of EU member states obtained from the GHSI and MAIRCA methods and the health safety performance scores obtained from the TOPSIS, BTA, WASPAS, COPRAS, EDAS, ARAS, MAUT, ROV, COCOSO, and GRA methods were also examined. According to the findings, there was a statistically significant and very high positive relationship between the GHSI and all methods except the MAUT method [17]. In another study, the 195 countries in the GHSI were evaluated using the PROMETHEE II and stochastic multicriteria acceptability analysis (SMAA) MCDMs based on the 2019 GHSI dataset. In that study, the indicators of the GHSI were determined as decision criteria and the 195 countries in the GHSI were taken as decision alternatives. At the end of the study, a three-stage multicriteria sequential cluster approach was proposed. It was revealed that the clusters formed within the scope of the GHSI and the clusters put forward within the scope of the study did not match each other exactly. For example, although countries such as the United States and the United Kingdom performed poorly during the COVID-19 pandemic, they were assigned to the cluster of most prepared countries in the pandemic according to the 2019 GHSI. However, countries such as Israel and Vietnam performed more effectively than the aforementioned countries in pandemic preparedness. In the multicriteria ranked cluster approach proposed in that study, countries such as Israel and Vietnam were assigned to the cluster of countries most prepared for the pandemic. At the end of the study, due to these inconsistencies, it was recommended to revise the pandemic preparedness classification of countries in the 2019 GHSI [18].

The use of the GHSI is limited to the subject area of global health security assessment. On the other hand, the BI-β [19], which was developed as an MCDM, can be used in solving multicriteria decision-making problems without sectoral discrimination. The first version of the BI was published in 2017 [20]. In 2023, the Beta version of the BI was published by shortening the application steps and the index was renamed as the BI-β. The BI-β method was first used in the financial assessment of the pharmaceutical sector in Türkiye [19]. On the other hand, studies in which the BI method was used have included the following:

- Financial performance analysis of organized industrial zones [20]; this study introduced the BI method.
- Evaluation of the financial performance of health institutions [21].
- Assessment of the performance of OECD countries in terms of the COVID-19 pandemic [22].
- Financial performance analysis for local governments [23].
- Financial performance analysis for renewable energy sector companies [24].

## 2. Materials and methods

In this study, the GHSI, which assesses global health security, and the BI-β method, developed as an MCDM, were compared in the context of the world’s countries. Thus, the feasibility of the BI-β method was demonstrated by testing it on GHSI datasets with the further aim of contributing to the methodological development of the GHSI. Additionally, an algorithm was written in the R programming language to increase the accessibility of the BI-β method on small-scale and especially large-scale datasets.

The dataset used in this study was the GHSI dataset, which allows for a comparative evaluation of countries. The GHSI dataset was obtained in csv format from the website https://www.ghsindex.org/, where GHSI reports are also published.^2^ There are 313 variables in the columns of the GHSI dataset, including country name, year, and indicators. The number of observations in the dataset is 122,070. The dataset was edited by applying data mining processes to it, and International Organization for Standardization Alpha-3 codes, regions, and subregions published by the United Nations were added to the dataset.^3^ After data mining, the number of rows in the dataset was 17,160 and the number of variables was 13.

In this study, 37 indicators from the GHSI were used for the comparison of the BI-β method and the GHSI. The categories and indicators were coded for ease of analysis (Table 1). The category codes consist of the abbreviation of the first letter of the English equivalent of the category name. The indicator codes consist of three letters: the first letter from the left indicates the category code, the second letter from the left indicates the category in which the indicator is included, and the third letter from the left indicates the order of the indicator in the respective category. These indicators are the decision criteria in the BI-β method.

The results obtained from the GHSI and the BI-β method do not necessarily imply that a country has or does not have global health security capacity. The BI-β provides a cross-sectional view of countries’ health security through publicly available data, as does the GHSI. Within the scope of this study, the decision alternatives are the countries included in the GHSI. The number of decision alternatives used in the BI-β method for 2019 and 2021 is 195.

Microsoft Excel (2018 version)^4^ was used to create decision matrices and tables, and the R programming language [25] was used to create analyses and maps. Specific to this study, a BI-β method application algorithm was written for the first time in R and BI-β analysis outputs were obtained. The obtained outputs were also compared with the outputs obtained in Microsoft Excel for a control. Nonparametric Spearman’s rank and Kendall’s Tau correlation tests were used to compare the score rankings obtained with the BI-β method and the GHSI. IBM SPSS Statistics 24 [26] was used for these statistical tests.

According to the 2021 GHSI report, the neutral weighting method was used to weight the indicators in calculating the GHSI scores for 2019 and 2021.^5^ In this study, a parallel approach was followed in order to make comparisons. For this purpose, 2019 and 2021 GHSI indicators were determined as the decision criteria in the BI-β method and the neutral weights were calculated for the decision criteria (Table 2). The indicator weights used in the BI-β method were calculated by proportioning GHSI neutral weighting coefficient values to the sum of GHSI neutral weighting coefficient values. Thus, in the new case, the sum of the weights used for the decision criteria in the BI-β method was reduced to 1 as required by the MCDM literature [27–37] (Table 2). In addition, since they are used in the BI-β method, the bestness criterion and ideal values of the decision criteria were also included. Ideal values were taken as the average of the values of each decision criterion and given by years (Table 2). In the literature, ideal values for the indicators or decision criteria in the GHSI have not been determined. In addition, in the GHSI study, the decision criteria were scored between 0 and 100. For these reasons, ideal values were calculated by averaging the values of each decision criterion. On the other hand, the main reason for maximizing the optimality criterion in the BI-β is that the answers to the lowest-level questions that constitute the scores of the decision criteria in the GHSI are usually dichotomous and structured according to Likert-type scales (Table 2). Subindicator scores are formed from questions, and indicator scores are formed from subindicator scores in the GHSI. Indicators are assigned a positive value between 0 and 100 according to the level of the health security preparedness of countries in the GHSI.

### 2.1. Application stages of the BI-β method

The application stages of the BI-β method, which was developed as an MCDM, are explained in detail in this section. The application stages of the method are as follows [19]:

Stage 1: Creating the decision matrix (DM)

The decision matrix (*X_ij_*) has criteria in rows and factors or alternatives in columns and is given in Eq. (1). The decision matrix is c × r dimensional, where c indicates columns and r indicates rows in the decision matrix.


(1) $$X_{ij}=\left[\begin{array}{cccc} x_{11} & x_{12} & \ldots & x_{1c}\\ x_{21} & x_{22} & \ldots & x_{2c}\\ \ldots & \ldots & \ldots & \ldots \\ x_{r1} & x_{r2} & \ldots & x_{rc} \end{array}\right]$$

Stage 2: Creating the difference matrix (DIFM)

In this step, the ideal values of the decision criteria are first determined. Ideal values can be continuous or discrete variables. Ideal values determined in the literature can be taken, or average, minimum, and maximum values can be taken according to the situation of the decision criteria. The determining factor here is the purpose and design of the research. In the first stage, after creating matrix *X_ij_*, the absolute value of the difference of the values of the alternatives belonging to each criterion in the columns of the matrix from the ideal values is obtained. If the ideal values in the literature are taken as a basis, set *L_j_* in Eq. (2), which shows the ideal value set of the criteria, is used.


(2) $$L_{j}=\{l_{1},l_{2},l_{3}....,l_{n}\}$$

Eq. (3) shows the relationship between the set of criterion ideal values *L_j_* and matrix *L_ij._*


(3) $$L_{ij}=\left[\begin{array}{cccc} x_{11} & x_{12} & \ldots & x_{1c}\\ x_{21} & x_{22} & \ldots & x_{2c}\\ \ldots & \ldots & \ldots & \ldots \\ x_{r1} & x_{r2} & \ldots & x_{rc} \end{array}\right]=\left[\begin{array}{c} l_{1}\\ l_{2}\\ \ldots \\ l_{r} \end{array}\right]$$

If there is no ideal value in the literature for the criteria, ideal values can be calculated from the arithmetic mean of the criteria values. In this case Eq. (4), which shows the arithmetic mean value of each criterion, is used. In Eq. (4), *X̄_j_* shows the set of ideal values obtained by the arithmetic mean of each criterion.


(4) $${\bar{X}}_{j}=\{{\bar{x}}_{1},{\bar{x}}_{2},{\bar{x}}_{3},\ldots ,{\bar{x}}_{n}\}$$

In Eq. (5), the relationship of the criterion ideal value set *X̄_j_* calculated over the arithmetic mean with matrix *X_ij_* is given.


(5) $$X_{ij}=\left[\begin{array}{cccc} x_{11} & x_{12} & \ldots & x_{1c}\\ x_{21} & x_{22} & \ldots & x_{2c}\\ \ldots & \ldots & \ldots & \ldots \\ x_{r1} & x_{r2} & \ldots & x_{rc} \end{array}\right]=\left[\begin{array}{c} {\bar{x}}_{1}\\ {\bar{x}}_{2}\\ \ldots \\ {\bar{x}}_{r} \end{array}\right]$$

The difference with respect to the ideal values is obtained by calculating the difference of each alternative’s criterion value from the ideal value of the relevant criterion, and Eq. (6) is used for this purpose. In the next steps of the method, this process ensures that the decision criteria are 0 and above, thus allowing for index values that will make the output positive.


(6) $$F_{ij}=\{\begin{array}{l} \left|x_{ij}-{\bar{x}}_{j}\right|\\ \left|x_{ij}-l_{j}\right| \end{array}$$

*X̄_j_* and *l_j_* are considered together or separately with the set of ideal criterion values to form the *F_ij_* equation. The *F_ij_* equation is then applied to the *X_ij_* matrix to obtain matrix *F_ij_* in Eq. (7). This operation shows the deviation of the criterion values of each alternative from the ideal values.


(7) $$F_{ij}=\left[\begin{array}{cccc} f_{11} & f_{12} & \ldots & f_{1c}\\ f_{21} & f_{22} & \ldots & f_{2c}\\ \ldots & \ldots & \ldots & \ldots \\ f_{r1} & f_{r2} & \ldots & f_{rc} \end{array}\right]$$

Stage 3: Creating the matching matrix (MM)

In this step, while forming the matching matrix, the criteria with maximum decision criteria direction are preserved in matrix *M_ij_* shown in Eq. (10). On the other hand, if the direction of the decision criteria is minimum, the absolute value of the ideal values of these criteria is calculated and the maximum value of each criterion’s value set is determined. The absolute value of this value is then taken by calculating its difference from the elements of the criteria value set. For the described operations, Eq. (8) shows the maximum values obtained according to the decision criteria. Eq. (9), which is formed according to this operation, shows the criteria value set ( $Y_{j}^{-}$). operates as in Eq. (10) to form matrix *M_ij_*.


(8) $$Y_{j}^{-}=\{{Max}_{i=0}f_{ij}\}$$

(9) $$Y_{j}^{-}=\{f_{1}^{-},f_{2}^{-},f_{3}^{-},......,f_{r}^{-}\}$$

(10) $$M_{ij}=\left[\begin{array}{cccc} y_{1}-f_{11} & y_{1}-f_{12} & \ldots & y_{1}-f_{1c}\\ y_{2}-f_{21} & y_{2}-f_{22} & \ldots & y_{2}-f_{2c}\\ \ldots & \ldots & \ldots & \ldots \\ y_{c}-f_{r1} & y_{c}-f_{r2} & \ldots & y_{c}-f_{rc} \end{array}\right]=\left[\begin{array}{cccc} m_{11} & m_{12} & \ldots & m_{1c}\\ m_{21} & m_{22} & \ldots & m_{2c}\\ \ldots & \ldots & \ldots & \ldots \\ m_{r1} & m_{r2} & \ldots & m_{rc} \end{array}\right]$$

Stage 4: Creating the logarithmic transformation matrix (LTM)

In this stage, an integer value of +1 is added to each element of matrix *M_ij_* by Eq. (11). The natural logarithm (*ln*) is then calculated to obtain matrix *Q_ij_* in Eq. (12).


(11) $$Q_{ij}=ln(m_{ij}+1)$$

(12) $$Q_{ij}=\left[\begin{array}{cccc} \text{ln }(m_{11}+1) & \text{ln }(m_{12}+1) & \ldots & \text{ln }(m_{1c}+1)\\ \text{ln }(m_{21}+1) & \text{ln }(m_{22}+1) & \ldots & \text{ln }(m_{2c}+1)\\ \ldots & \ldots & \ldots & \ldots \\ \text{ln }(m_{r1}+1) & \text{ln }(m_{r2}+1) & \ldots & \text{ln }(m_{rc}+1) \end{array}\right]=\left[\begin{array}{cccc} q_{11} & q_{12} & \ldots & q_{1c}\\ q_{21} & q_{22} & \ldots & q_{2c}\\ \ldots & \ldots & \ldots & \ldots \\ q_{r1} & r_{r2} & \ldots & q_{rc} \end{array}\right]$$

If the decision criteria are to be weighted, the weighting operation is completed in this step. For this, the elements of matrix *Q_ij_* are multiplied by weight coefficients (*w_ij_*) to obtain the weighted decision matrix (*A_ij_*) in Eq. (13), where *W* = {*w*_1_,*w*_2_,*w*_3_,...,*w_n_*}denotes the set of weight coefficients of the criteria and $\sum_{i=1}^{n}w_{i}=1$.


(13) $$A_{ij}=\left[\begin{array}{cccc} w_{1}\times q_{11} & w_{1}\times q_{12} & \ldots & w_{1}\times q_{1c}\\ w_{2}\times q_{21} & w_{2}\times q_{22} & \ldots & w_{2}\times q_{2c}\\ \ldots & \ldots & \ldots & \ldots \\ w_{n}\times q_{r1} & w_{n}\times q_{r2} & \ldots & w_{n}\times q_{rc} \end{array}\right]=\left[\begin{array}{cccc} a_{11} & a_{12} & \ldots & a_{1c}\\ a_{21} & a_{22} & \ldots & a_{2c}\\ \ldots & \ldots & \ldots & \ldots \\ a_{r1} & a_{r2} & \ldots & a_{rc} \end{array}\right]$$

Stage 5: Determining the index reference values (IRVs)

In this step, the maximum of the set of criteria values of alternatives for each criterion represents the index reference value (*R_r_*) of the relevant criterion. Eq. (14) shows the maximum alternative value by criterion and Eq. (15) shows the maximum set of alternative values by criterion. The number of elements in the IRV set (*R_ri_*) is equal to the number of decision criteria (*C_n_*) and equal to a matrix of size *C_n_* × 1. This matrix contains the maximum values of alternatives according to the criterion.


(14) $$R_{r}=\{max_{i=0}a_{ij}\}$$

(15) $$R_{ri}=\{a_{11},a_{12},a_{13},......,a_{1n}\}$$

On the other hand, elements in the set of criteria values of alternatives in the matrix are given in Eq. (16).


(16) $$A_{ci}=\{a_{11},a_{12},a_{13},......,a_{rc}\}$$

Stage 6: Calculating the BI-β scores

In the fifth stage, the index reference score (*b_ri_*) in Eq. (17) is calculated by taking the sum of elements in the set of IRVs determined for each criterion. The in-class score (*t_ci_*) of each alternative is calculated using Eq. (18) over matrix *A_ij_* if weighting is applied and matrix *Q_ij_* if weighting is not applied *t_ci_* shows the sum of criteria values of the alternatives in columns of the matrix. Eq. (19) shows the BI-β score obtained by dividing the index reference score (*b_ri_*) by the in-class score (*t_ri_*) of alternatives and multiplying it by 100.


(17) $$b_{ri}=\Sigma_{i=1}^{n}R_{ri}$$

(18) $$t_{ci}=\sum_{i=1}^{n}A_{ci}$$

(19) $$0\leq BE-\beta \leq 100\;\;\;\text{and }\;\;BE-\beta =\frac{t_{ci}}{b_{ri}}\times 100$$

Specific to this study, an application algorithm for the BI-β method was written for the first time in R (Appendix 1) and BI-β analysis outputs were obtained. The libraries used in R for reading the decision matrices to the bbeta function, printing the findings obtained from the bbeta function to a Microsoft Excel workbook with the xlsx extension, and creating maps from the findings were as follows: “dplyr” [38], “ggplot2” [39], “ggthemes” [40], “ggpubr” [41], “giscoR” [42], “forcats” [43], “magrittr” [44], “openxlsx” [45], “readxl” [46], “rmarkdown” [47–49], “rnaturalearth” [50], “sf” [51], “sp” [52,53], “tibble” [54], “tidyr” [55], “tmap” [56], and “viridis” [57].

### 2.2. Statistical tests

In this section, the nonparametric statistical tests used to test whether the rankings obtained from the BI-β and GHSI are statistically different from each other are introduced.

Spearman’s rank and Kendall’s Tau correlation tests are among the most widely used nonparametric tests for comparing whether two rankings are statistically different from each other [58]. There are studies in which these tests are used both in the comparison of MCDMs and in the comparison of indices and MCDMs. Spearman’s rank and Kendall’s Tau correlation tests are frequently used to compare the rankings obtained from MCDMs [16,19,59–66] and to compare indices and MCDMs in the health sector [17]. For these reasons, Kendall’s Tau and Spearman’s rank correlation tests were used to determine whether there was a statistically significant difference between the rankings obtained from the BI-β and GHSI.

Spearman’s rank and Kendall’s Tau correlation coefficients measure the relationship and degree of agreement between two quantitative variables. Spearman’s rank correlation coefficient is denoted by *r_s_* and Kendall’s Tau correlation coefficient is denoted by *τ*. The correlation coefficients, which can take values between –1 and +1, are interpreted as follows [67–70]: a correlation coefficient value of 0 indicates that there is no monotonic relationship between two ranking groups, while a coefficient value of +1 or –1 indicates a perfect monotonic relationship.

For Spearman’s rank correlation (*r_s_*) [67,71,72], as given in Eq. (20), *n* is the number of observations in the rankings, *S_s_* is the sum of squared differences, and *d_i_* is the difference between paired observation rankings.


(20) $$S_{s}=\sum_{i=1}^{n}{\left(d_{i}\right)}^{2},r_{s}=1-\frac{6S_{s}}{n^{3}-n}$$

For Kendall’s Tau correlation coefficient [64,69,73,74], as given in Eq. (21) , *n* indicates the number of element pairs, *p* indicates the number of compatible pairs, and *q* indicates the number of incompatible pairs.


(21) $$\tau =2\times \frac{p-q}{n(n-1)}$$

The null hypothesis (H_0_) and alternative hypothesis (H_A_) in Spearman’s rank and Kendall’s Tau correlation tests used to determine whether the rankings obtained from the BI-β method and GHSI are statistically different from each other are as follows:

- H_0_: There is no monotonic relationship between BI-β and GHSI scores.
- H_A_: There is a monotonic relationship between BI-β and GHSI scores.

## 3. Results

In this section, the BI-β method is used to evaluate 195 countries included in the GHSI in terms of global health security. Whether the rankings obtained from the GHSI and BI-β method are statistically significant or not is then tested with Spearman’s rank and Kendall’s Tau correlation tests, and the findings of the tests are presented.

The sixth and final step of the BI-β method is the calculation of BI-β scores. In this step, the BI-β score is obtained by proportioning the in-class score of each alternative to the sum of the IRVs and multiplying it by 100. The in-class score (*t_ci_*) represents the sum of criteria values of the alternatives in a weighted logarithmic transformation matrix. Table 3 shows the top 33 countries with the highest scores in 2021 according to the BI-β method. In addition, in order to make a holistic evaluation, the BI-β scores for 2019 are also presented according to the countries with 2021 rankings (Table 3). The BI-β scores of the world’s countries in terms of global health security are as follows: in 2021, the top three best-performing alternatives in terms of global health security were the United States (BI-β_2021_ = 85.46), Australia (BI-β_2021_ = 82.53), and the United Kingdom (BI-β_2021_ = 82.29). In 2019, the United States (BI-β_2019_ = 85.44) and Australia (BI-β_2019_ = 81.69) had the same ranks as in 2021, while the United Kingdom (BI-β_2019_ = 76.63) fell to 9th place.

For comparison purposes, a world map showing the BI-β scores of all countries in 2019 and 2021 is given (Figure). When the BI-β results are evaluated, Nigeria (NGR) was the country with the highest increase in 2021 compared to 2019 (BI-β_2019_ = 54.41, BI-β_2021_ = 62.18, increase = 7.77 BI-β). On the other hand, the country with the highest decrease in 2021 compared to 2019 was Djibouti (DJI) (BI-β_2019_ = 65.39, BI-β_2021_ = 61.58, decrease = 3.81 BI-β).

Spearman’s rank and Kendall’s Tau correlation tests were conducted to test whether the difference between the worldwide score rankings obtained from the BI-β and GHSI were statistically significant by years (Table 4). The findings revealed a statistically significant positive weak monotonic relationship between BI-β and GHSI rankings in 2019 according to Spearman’s rank correlation (r_s_(193) = 0.259, p = 0.000, N = 195). Similarly, a statistically significant positive weak relationship was observed between BI-β and GHSI rankings in 2019 by Kendall’s Tau correlation (τ = 0.179, p = 0.000, N = 195). For these reasons, the H_0_ hypothesis was rejected for both correlation tests. Similar to the results of 2019, a statistically significant positive weak relationship was found between BI-β and GHSI rankings in 2021 by Spearman’s rank correlation (r_s_(193) = 0.252, p = 0.000, N = 195) and Kendall’s Tau correlation (τ = 0.166, p = 0.000, N = 195).

## 4. Discussion

The findings obtained from the GHSI and the BI-β method developed as an MCDM were analyzed and supported by studies in the literature. For the most up-to-date results, evaluations were made according to GHSI rankings for 2021 based on the BI-β and GHSI findings for 2021.

When the findings of the world’s countries for 2021 were analyzed by rank correlation tests, a statistically significant positive weak monotonic relationship between BI-β and GHSI rankings was observed. In a similar study [16], the performances of the health systems of 195 countries were analyzed using six indicators from the health dimension of the 2009 GHSI with MCDMs. In that study, the relationship between the rankings obtained from the modified VIKOR, TOPSIS, GRA, and COPRAS methods was measured with Spearman’s rank correlation coefficient. According to the findings, there was no statistically significant monotonic relationship between the modified VIKOR and other methods [16].

In 2021, the number of countries with the same BI-β and GHSI rankings was 6 out of 195 countries, and these countries were as follows with their rankings: United States (1), Australia (2), Canada (4), France (14), Mexico (25), Namibia (134), and Belize (139). There were no matches between the BI-β and GHSI rankings of other countries of the world. On the other hand, the rankings of 33 countries out of 195 countries were the same due to the GHSI scoring system in the 2021 GHSI. According to the BI-β method, there were no countries with the same scores and rankings. From this point of view, it is seen that the 2021 GHSI does not clearly reveal the rankings of the world’s countries with full differentiation. One reason for this is the use of an arbitrary neutral weighting method in the weighting of the categories of the GHSI [7]. Another reason for the difference is that although the same weighting method is used for the comparison of the BI-β method and the GHSI, the theoretical concept of the BI-β method is different from that of the GHSI [16].

The discrepancy between GHSI scores and the actual performances of countries during the COVID-19 pandemic is another point of criticism for the GHSI. In a study in which the effectiveness of COVID-19 vaccine use in OECD countries was estimated with the GHSI, Spearman’s rank correlation coefficient was not statistically significant [10]. The rank correlation coefficient was also close to 0. In that study, it was stated that although Israel was the country with the highest proportion of the population fully vaccinated against COVID-19 within 2 months after the launch of the global vaccine, it ranked 34th in terms of pandemic preparedness in the GHSI. In another study with similar findings, the epidemiological wavelength method was developed to measure the size of the COVID-19 outbreak [75]. The size of the pandemic in OECD member countries was revealed using the epidemiological wavelength method by years. According to the findings, the United States was the country with the largest pandemic size in 2020, 2021, and 2022. However, in 2019 and 2020, the United States was the best country among 195 countries in the GHSI in terms of global health security and pandemic preparedness. In another study, the results of the 2019 GHSI were compared with results of the multicriteria ranked cluster approach proposed in this study [18]. It was found that the country clusters in the GHSI and the clusters put forward within the scope of that study did not match each other exactly. For example, although countries such as the United States and the United Kingdom performed poorly during the COVID-19 pandemic, they were assigned to the cluster of most prepared countries for a pandemic by the 2019 GHSI. However, countries such as Israel and Vietnam performed more effectively in pandemic preparedness than these countries. Therefore, with the proposed multicriteria ordered cluster approach, countries such as Israel and Vietnam were assigned to the cluster of most prepared countries for the pandemic [18]. In summary, higher scores of countries in the GHSI do not mean that they responded optimally to the COVID-19 outbreak [15].

## 5. Conclusion

The GHSI, structured as a composite index, monitors the development processes of countries by revealing their ability to prepare for and respond to infectious disease outbreaks that may become epidemics or global pandemics, such as the COVID-19 pandemic. In this way, it is aimed to transparently assess global health security and improve the international capability to combat infectious disease threats that may lead to national and international outbreaks. The first report on the GHSI was published in 2019 and the second edition was published in 2021 with an expansion of its scope with additional indicators and questions.

In the present study, the GHSI, covering the years 2019 and 2021, and the BI-β, developed as an MCDM, were compared in the context of the global health security preparedness of the world’s countries. The comparison was made according to the decision criteria and the decision alternative countries included in the GHSI. In this way, the feasibility of the BI-β method was demonstrated by testing it on the GHSI dataset and suggestions were made to contribute to the methodological development of the GHSI. An application algorithm was also presented for academics and field workers who will implement the BI-β method. With the application algorithm, it is possible to produce instant solutions with ease of implementation for small-scale and especially large-scale datasets. The BI-β method, like the BI method, can be used to solve multicriteria decision problems in any sector. In the field of healthcare, where big data and large datasets are generated, the BI-β method has the potential to be applied in a number of ways using the application algorithm. These include evaluations of the financial performance of hospitals, resolution of problems associated with selection of hospital sites, assessment of quality of units within hospitals, the purchasing of materials and equipment for hospitals, evaluations of marketing strategies in the health sector, and evaluations of primary and secondary healthcare facilities.

The findings obtained using the BI-β method and the methodological framework of the BI-β method are expected to provide a different perspective on the methodological framework of the GHSI. In this way, it is anticipated that the present study will contribute to the methodological development of the GHSI.

When the countries of the world were compared in terms of global health security preparedness by the BI-β method and GHSI, a statistically significant positive weak monotonic relationship between BI-β and GHSI rankings was found according to rank correlation tests. From this finding, it can be understood that the rankings obtained from the BI-β method and GHSI are relatively consistent. When the countries of the world are analyzed using the BI-β and GHSI, the necessary steps to be taken to increase the efficiency of the GHSI can be summarized as follows:

- The monotonic levels of association between the GHSI and BI-β method do not reveal causal relationships between health system capacities and observed health outcomes. Therefore, pre- and postpandemic in-country assessments and further analyses are needed to establish causal relationships.
- An analysis of the GHSI methodology reveals that the indicators are arbitrarily assigned neutral weights. This constitutes another problem area of the GHSI. Therefore, the weighting process should be reconsidered objectively and weighting processes should be explained together with their reasons. In this way, the GHSI methodology may be more widely accepted.
- In some low- and middle-income countries, important information and documents are not accessible or publicly available. Therefore, countries with better skills and capacities may score lower in the GHSI, distorting the results. For this reason, excluding these countries from evaluation in the GHSI and evaluating only the countries with full data availability may contribute to the efficiency of the GHSI.
- Although the GHSI is a valid measurement tool for assessing health security on a global scale, it has potential shortcomings in the measurement of global health security. Since the GHSI was conceptualized based on a narrow and technical framework, the social and political determinants of public health are not taken into account in the GHSI. Including these determinants within the scope of the GHSI could make significant contributions to the GHSI because improving and sustaining the health capacities of countries in fighting against epidemics requires significant investments and strong political will. Moreover, since the structure of a health system directly affects a country’s health security and preparedness for pandemics, these factors should be taken into account and included in the GHSI.
- The lack of definitions of indicators in the publicly published 2019 and 2021 reports of the GHSI constitutes a deficiency in itself. It is believed that eliminating this deficiency will contribute to a better understanding and wider acceptance of the GHSI on a global scale.
- In order for the GHSI to produce more effective and measurable results, it needs data and, more importantly, quality data flow. Therefore, countries should be more transparent and willing to share data.

In this study, the results obtained from the BI-β method were limited to 37 indicators identified as decision criteria within the scope of the GHSI and 195 countries identified as decision alternatives. Therefore, the global health security and pandemic preparedness of the countries were evaluated based on these decision criteria and decision alternatives. This is a limitation of the study. The results obtained from the GHSI and BI-β method do not necessarily mean that countries have or do not have global health security capacity. In other words, a high GHSI score does not necessarily mean that a country had an optimal response to the COVID-19 pandemic. At the same time, since the GHSI and BI-β reveal the cross-sectional health security outlooks of countries at a certain point in time through publicly available data, the findings obtained from these methods should not be used as a forecasting tool for the future. Furthermore, the data used in this study only cover the GHSI report periods of 2019 and 2021, and the COVID-19 outbreak start times for each country were not the same in 2019 and 2021. This may have caused differences between these two years in the decision alternative countries evaluated in terms of global health security. These observations reflect assumptions and limitations of the present study.

Since the rank correlation coefficients between the GHSI and BI-β method do not reveal causal relationships between health system capacities and observed health outcomes, in-country assessments and analyses before and after outbreaks are needed to reveal causal relationships. It can be suggested that decision-makers would then gain more insights and make more reliable public health decisions.

The inclusion of social and political determinants of public health in the GHSI would allow for a more comprehensive assessment of global health security for countries and the making of more reliable decisions. Therefore, it is recommended to take these determinants into account. Instant solutions can be produced in solving multicriteria decision-making problems with large-scale datasets using the BI-β application algorithm written in the R environment. By utilizing the algorithm in solving other MCDM problems, especially in public health, analysis outputs can be evaluated from a wider perspective in future studies.

Since the main focus of this study was to demonstrate the relatively new BI-β method and its implementation on the GHSI dataset, future studies may compare the BI-β method with other MCDMs and evaluate the BI-β method from a broader perspective.

## Figures and Tables

**Figure:**
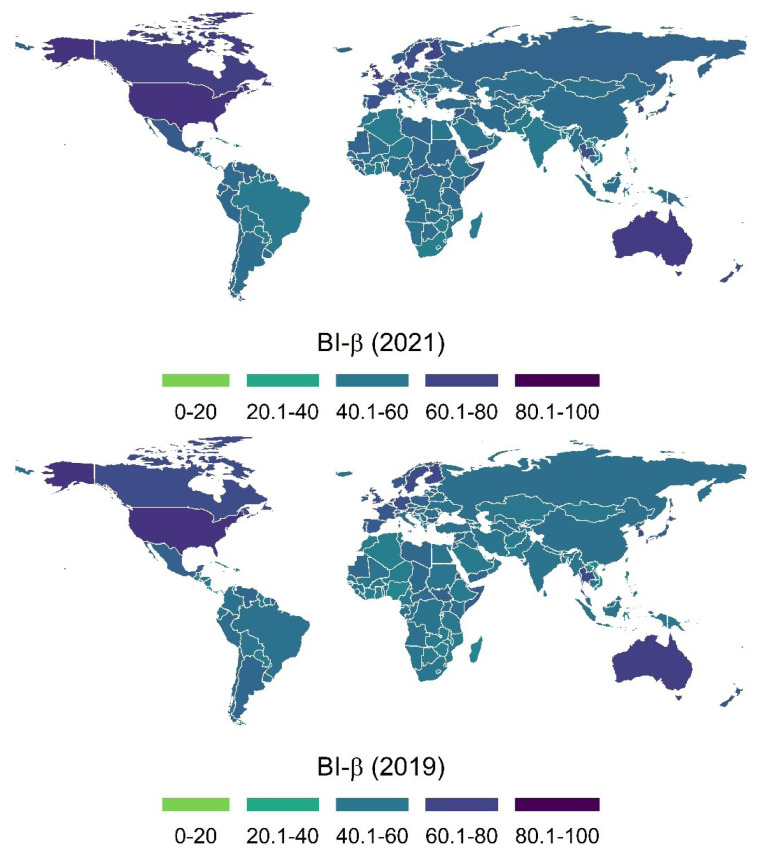
BI-β scores of the world’s countries, 2019–2021.

**Table 1:** Categories and indicators in GHSI.

Category	Code	Indicator	Code
Prevent	p	Antimicrobial resistance (AMR)	p11
		Zoonotic disease	p12
		Biosecurity	p13
		Biosafety	p14
		Dual-use research and culture of responsible science	p15
		Immunization	p16
Detect	d	Laboratory systems strength and quality	d21
		Laboratory supply chains	d22
		Real-time surveillance and reporting	d23
		Surveillance data accessibility and transparency	d24
		Case-based investigation	d25
		Epidemiology workforce	d26
Respond	r	Emergency preparedness and response planning	r31
		Exercising response plans	r32
		Emergency response operation	r33
		Linking public health and security authorities	r34
		Risk communication	r35
		Access to communications infrastructure	r36
		Trade and travel restrictions	r37
Health	h	Health capacity in clinics, hospitals, and community care centers	h41
		Supply chain for health system and healthcare workers	h42
		Medical countermeasures and personnel deployment	h43
		Healthcare access	h44
		Communications with healthcare workers during a public health emergency	h45
		Infection control practices	h46
		Capacity to test and approve new medical countermeasures	h47
Norms	n	IHR reporting compliance and disaster risk reduction	n51
		Cross-border agreements on public health and animal health emergency response	n52
		International commitments	n53
		JEE and PVS	n54
		Financing	n55
		Commitment to sharing of genetic & biological data & specimens	n56
Risk	r	Political and security risk	r61
		Socioeconomic resilience	r62
		Infrastructure adequacy	r63
		Environmental risks	r64
		Public health vulnerabilities	r65

**Table 2:** Weight coefficients in GHSI and BI-β.

Category	Code	Neutral weight		Optimality criterion	Ideal value	
		GHSI	BI-β		2019	2021
Prevent	p11	0.167	0.028	Maximum	43.412	45.335
	p12	0.167	0.028	Maximum	24.667	19.825
	p13	0.167	0.028	Maximum	18.168	18.654
	p14	0.167	0.028	Maximum	20.769	20.897
	p15	0.167	0.028	Maximum	2.649	2.649
	p16	0.167	0.028	Maximum	64.744	63.333
Detect	d21	0.167	0.028	Maximum	39.423	44.872
	d22	0.167	0.028	Maximum	13.590	15.897
	d23	0.167	0.028	Maximum	35.769	34.551
	d24	0.167	0.028	Maximum	33.687	34.728
	d25	0.167	0.028	Maximum	8.269	16.923
	d26	0.167	0.028	Maximum	45.641	46.538
Respond	r31	0.143	0.024	Maximum	21.052	30.366
	r32	0.143	0.024	Maximum	6.282	21.090
	r33	0.143	0.024	Maximum	25.110	26.989
	r34	0.143	0.024	Maximum	22.564	22.051
	r35	0.143	0.024	Maximum	52.844	57.886
	r36	0.143	0.024	Maximum	66.016	65.704
	r37	0.143	0.024	Maximum	97.308	38.974
Health	h41	0.143	0.024	Maximum	21.584	30.029
	h42	0.143	0.024	Maximum	26.294	28.546
	h43	0.143	0.024	Maximum	9.231	10.256
	h44	0.143	0.024	Maximum	54.808	55.218
	h45	0.143	0.024	Maximum	10.513	10.769
	h46	0.143	0.024	Maximum	39.487	40.513
	h47	0.143	0.024	Maximum	44.231	45.128
Norms	n51	0.167	0.028	Maximum	62.051	58.462
	n52	0.167	0.028	Maximum	50.000	50.000
	n53	0.167	0.028	Maximum	53.223	56.131
	n54	0.167	0.028	Maximum	17.692	18.718
	n55	0.167	0.028	Maximum	33.421	35.213
	n56	0.167	0.028	Maximum	68.236	68.407
Risk	r61	0.200	0.033	Maximum	58.718	58.103
	r62	0.200	0.033	Maximum	61.158	60.865
	r63	0.200	0.033	Maximum	48.976	50.215
	r64	0.200	0.033	Maximum	55.514	54.714
	r65	0.200	0.033	Maximum	55.061	55.313
**Sum**		**6.000**	**1.000**			

**Table 3:** BI-β scores of world countries, 2019–2021.

Code	Alternative	2021				2019			
		a t_ci_	b b_ri_	BI-β	Rank	a t_ci_	b b_ri_	BI-β	Rank
USA	United States of America	3.46	4.05	85.46	1	3.5	4.09	85.44	1
AUS	Australia	3.35	4.05	82.53	2	3.34	4.09	81.69	2
GBR	United Kingdom	3.34	4.05	82.29	3	3.14	4.09	76.63	9
CAN	Canada	3.30	4.05	81.48	4	3.16	4.09	77.22	8
FIN	Finland	3.23	4.05	79.69	5	3.19	4.09	78.02	3
KOR	South Korea	3.19	4.05	78.72	6	3.13	4.09	76.59	10
DEU	Germany	3.18	4.05	78.54	7	3.12	4.09	76.2	12
SVN	Slovenia	3.15	4.05	77.66	8	3.13	4.09	76.45	11
NZL	New Zealand	3.11	4.05	76.75	9	3	4.09	73.38	14
DNK	Denmark	3.09	4.05	76.28	10	3.16	4.09	77.27	7
THA	Thailand	3.09	4.05	76.19	11	3.18	4.09	77.62	4
SWE	Sweden	3.09	4.05	76.17	12	3.17	4.09	77.39	6
BGR	Bulgaria	3.03	4.05	74.65	13	2.97	4.09	72.47	17
FRA	France	3.02	4.05	74.53	14	2.99	4.09	73.06	15
NLD	Holland	3.01	4.05	74.18	15	3.17	4.09	77.41	5
CHE	Switzerland	3.00	4.05	73.99	16	3.05	4.09	74.65	13
GNB	Guinea-Bissau	3.00	4.05	73.94	17	2.93	4.09	71.48	20
ARM	Armenia	3.00	4.05	73.9	18	2.97	4.09	72.57	16
ESP	Spain	2.98	4.05	73.4	19	2.92	4.09	71.37	21
IRL	Ireland	2.96	4.05	73.09	20	2.9	4.09	70.75	24
AUT	Austria	2.95	4.05	72.88	21	2.92	4.09	71.28	22
YEM	Yemen	2.95	4.05	72.85	22	2.75	4.09	67.25	40
ERI	Eritrea	2.95	4.05	72.74	23	2.8	4.09	68.35	33
SOM	Somalia	2.94	4.05	72.58	24	2.93	4.09	71.53	19
MEX	Mexican	2.94	4.05	72.57	25	2.85	4.09	69.68	27
NOR	Norway	2.93	4.05	72.28	26	2.96	4.09	72.37	18
SYR	Syria	2.93	4.05	72.18	27	2.78	4.09	68.04	35
LVA	Latvia	2.92	4.05	71.98	28	2.9	4.09	70.84	23
PRK	North Korea	2.87	4.05	70.88	29	2.75	4.09	67.19	41
LTU	Lithuania	2.87	4.05	70.79	30	2.65	4.09	64.67	67
BDI	Burundi	2.87	4.05	70.69	31	2.73	4.09	66.8	44
JPN	Japan	2.86	4.05	70.55	32	2.86	4.09	70.01	25
SGP	Singapore	2.85	4.05	70.41	33	2.86	4.09	69.97	26

a **t_ci_:** in-class score,

b **b_ri_:** sum of index reference values.

**Table 4:** BI-β and GHSI correlation tests, world countries.

	Kendall tau			Spearman’s rank		
Year	N	τ	p	N	r_s_	p
2019	195	0.179**	0.000	195	0.259**	0.000
2021	195	0.166**	0.001	195	0.252**	0.000

** The correlation is statistically significant at the 0.01 level (2-tailed).

## Footnotes

1 World Health Organization (2000). A Framework for Global Outbreak Alert and Response. World Health Organization [online]. Website https://apps.who.int/iris/handle/10665/66789 [accessed 17 July 2023].

2 GHSI (2023). 2021 GHSI Index [online]. Website https://www.ghsindex.org/wp-content/uploads/2022/04/2021-GHS-Index-April-2022.csv [accessed 5 April 2023].

3 International Organization for Standardization (2020). Country Codes (ISO 3166-1 and ISO 3166-3) [online]. Website https://www.iso.org/glossary-for-iso-3166.html [accessed 23 April 2023]; United Nations Department of Economic and Social Affairs (2023). Methodology: Standard Country or Area Codes for Statistical Use (M49) [online]. Website https://unstats.un.org/unsd/methodology/m49/overview/ [accessed 11 April 2023].

4 Microsoft Corporation (2018). Microsoft Excel [online]. Website https://office.microsoft.com/excel [accessed 5 April 2023].

5 Economist Impact (2021). Global Health Security Index Methodology [online]. Website https://www.ghsindex.org/wp-content/uploads/2021/11/2021_GHSindex_Methodology_FINAL.pdf [accessed 11 April 2023].

## Appendix 1 Application Algorithm of BI-β Method in R

bbeta() function, which is application algorithm of BI-β method, was written using R programming language. Instantaneous BI-β findings can be produced by bbeta() function in large data sets. The code block of bbeta() function written in R is given below:

bbeta <- **function**(dm=as.data.frame(NULL), dc=NULL, wc=1, iv=NULL, a=NULL, cr=NULL){
     colnames(dm)=a; rownames(dm)=cr
     dm2<-dm
     dc1<-ifelse(dc==”max”, 1, 0)
     dm3<-dm
     dm4<-dm
     dm5<-dm
     dc2<-dc1
     **for** (r **in** 1:nrow(dm))
                 **for** (c **in** 1:ncol(dm))
                       dm2[r,c] <- abs(dm[r,c]-iv[r])
     rownames(dm2)<-cr
     **for** (r **in** 1:nrow(dm2))
               **for** (c **in** 1:ncol(dm2))
                  **if** (dc2[r]) {
                         dm3[r,c] <- dm2[r,c]
                             } **else** {
                      dm3[r,c]<-abs(max(dm2[r,]) - abs(dm2[r,c]))
     }
     rownames(dm3)<-cr
     dm4<-logb(dm3 + 1)
     dm5<-dm4*wc
     rownames(dm5)<-cr
     irv<-as.matrix(apply(dm5, 1, max))
     rownames(irv)<-cr
     colnames(irv)<-c(“Index Reference Values”)
     is<-as.matrix(apply(dm5,2, sum))
     colnames(is)<-c(“In-class Score”)
     bi_beta<-as.matrix((is/sum(irv))*100)
     k<-as.matrix(bi_beta[order(bi_beta[,1],decreasing=T),])
     rank<-as.matrix(1:nrow(bi_beta))
     bi_beta<-as.matrix(cbind(k, rank))
     colnames(bi_beta)<-c(“BI-Beta”, “Rank”)
     **return**(list(Decision_Matrix=as.matrix(dm), Stage_2=as.matrix(dm2), Stage_3=as.matrix(dm3), Stage_4=as.
matrix(dm4), Weighted_Matrix=as.matrix(dm5), IRV=irv, In_class_Score=is, “BI_Beta”=bi_beta))
     }

The variables defined in bbeta() function are as follows:

- dm is defined as a decision matrix. The rows of dm contain decision criteria and columns contain alternatives.
- The direction of criterion determined as dc is defined as maximum or minimum in vector format. In other words, maximum is equal to “max” and minimum is equal to “min”.
- wc shows weight levels of decision criteria. wc should be defined as a vector. The default value of wc is set to 1. When wc is equal to 1, it indicates that decision criteria are not weighted.
- iv shows ideal values of decision criteria. iv can be determined in three different ways. Firstly, iv can be defined according to direction of the criterion, that is, according to whether decision criterion is maximum or minimum. Secondly, ideal values can be determined according to average of decision criteria. Finally, decision maker can determine ideal values according to literature review or expert opinions.
- a is a vector containing names of decision alternatives.
- cr is a vector containing names of decision criteria.

The outputs that can be obtained from bbeta() function are defined in list format within the function. The outputs defined in list format are as follows:

- Decision_Matrix shows the first stage decision matrix (DM).
- Stage_2 shows the second stage difference matrix (DIFM).
- Stage_3 shows the third stage matching matrix (MM).
- Stage_4 shows the fourth stage logarithmic transformation matrix (LTM)
- Weighted_Matrix indicates the weighted logarithmic transformation matrix (WLTM).
- IRV stands for index reference values (IRV).
- In_Class_Score indicates in-class score of decision alternatives.
- BI_Beta shows BI-β scores of decision alternatives and ranking of decision alternatives.
